# Tinnitus-associated cognitive and psychological impairments: a comprehensive review meta-analysis

**DOI:** 10.3389/fnins.2024.1275560

**Published:** 2024-02-08

**Authors:** Dong Yang, Dan Zhang, Xinmiao Zhang, Xin Li

**Affiliations:** ^1^Department of Otorhinolaryngology, Tianjin Medical University General Hospital, Tianjin, China; ^2^Department of Psychology, Tsinghua University, Beijing, China; ^3^Department of Otorhinolaryngology, Beijing Tsinghua Changgung Hospital, Beijing, China; ^4^School of Clinical Medicine, Tsinghua University, Beijing, China

**Keywords:** tinnitus, cognitive and psychological impairments disorders, Alzheimer's disease, dementia, poor sleep quality, anxiety, learning and auditory attention, attention deficit disorder

## Abstract

**Background:**

Tinnitus is strongly associated with an increased risk of cognitive disabilities. The findings of this research will provide valuable support for future investigations aimed at determining the correlation between tinnitus and the risk of cognitive impairments.

**Objectives:**

We investigated the potential correlation between tinnitus and the risk of various cognitive impairments, such as dementia, compromised learning attention, anxiety, depression, and insomnia. The study examined this relationship collectively and by categorizing the data based on different age groups.

**Methods:**

We compiled data from case–control studies and cohort studies obtained from reputable databases such as PubMed, Cochrane Library, and Embase. To minimize potential bias, two reviewers independently assessed the selected articles. After extracting the data, we calculated the pooled odds ratios (ORs) using a random-effects model.

**Results:**

Seventeen relevant studies, comprising an adult population, were included in this analysis. Pooled estimated outcomes revealed a strong association between tinnitus and an elevated risk of dementia-compromised learning, auditory attention, anxiety, depression, and poor sleep quality (P<0.05). Furthermore, the pooled analysis stratified by age demonstrated that patients aged above 60 years, in comparison to those aged 18 to 60 years, exhibited more significant outcomes in relation to the progression of cognitive impairments.

**Conclusion:**

Tinnitus has the potential to increase the risk of cognitive impairments. Moreover, geriatric patients aged above 60 shows a higher susceptibility to developing cognitive disabilities compared to their younger counterparts.

## 1 Introduction

Tinnitus is a common auditory symptom characterized by the perception of sound in the absence of any external source. It affects millions worldwide and can vary in intensity and duration (Baguley et al., 2013). While the underlying mechanisms of tinnitus are not fully understood, it is believed to arise from complex interactions between the auditory system and the brain (Kompis et al., 2004). Tinnitus can have a significant impact on individuals' quality of life, leading to emotional distress, sleep disturbances, and concentration difficulties. Various factors contribute to its development, including noise exposure, age-related hearing loss, and certain medical conditions (Han et al., 2009). Although there is no cure for tinnitus, management strategies such as sound therapy, cognitive-behavioral therapy, and medication can help to alleviate its symptoms and improve overall wellbeing (Ost, 2014).

Tinnitus has been associated with various underlying medical conditions and diseases. Some examples include age-related hearing loss, ototoxic medications, temporomandibular joint (TMJ) disorders, Meniere's disease, acoustic neuroma, and cardiovascular disorders such as hypertension (Savage and Waddell, 2014). Additionally, mental health conditions such as anxiety, depression, and post-traumatic stress disorder (PTSD) have also been linked to tinnitus (Pattyn et al., 2016). Understanding these associations can aid in the diagnosis and management of tinnitus, as treating the underlying condition may help to alleviate tinnitus symptoms.

Tinnitus has been found to be associated with various cognitive disorders. Studies have shown a higher prevalence of tinnitus in individuals with cognitive impairments such as memory problems and mild cognitive impairment (Jafari et al., 2019; Chu et al., 2020). Cognitive decline and tinnitus may share common underlying mechanisms, including neuroinflammation, oxidative stress, and neurotransmitter imbalances (Paciello et al., 2023). Tinnitus can also contribute to cognitive difficulties, as the constant perception of sound can interfere with attention, concentration, and memory processes (Lan et al., 2020). Furthermore, the emotional distress and psychological impact of tinnitus can exacerbate cognitive symptoms in individuals with existing cognitive disorders. Tinnitus has a significant association with sleep disturbances and insomnia (Alster et al., 1993). The perception of tinnitus can disrupt sleep patterns, making it difficult for individuals to fall asleep or maintain sleep throughout the night. Sleep deprivation and poor sleep quality can further exacerbate tinnitus symptoms, creating a vicious cycle that negatively impacts overall wellbeing (Gallo et al., 2023).

This meta-analysis aimed to investigate the association between tinnitus and various cognitive impairments, including dementia, Parkinsonism, depression, learning and auditory attention, stress, anxiety, and poor sleep quality. Meta-analyses are research studies that analyze and combine data from multiple studies to draw overall conclusions, providing a broader understanding of the topic being studied.

## 2 Materials and methods

### 2.1 Search methodology

Different search engines were used to collect data, including the Cochrane Library, PubMed, and Embase databases. The Medical Subject Headings (MeSH) terms included “tinnitus,” “cognitive impairments”, “tinnitus-induced cognitive impairments”, “mild cognitive disabilities,” “dementia,” “tinnitus induced poor sleep,” “tinnitus-induced altered learning,” and “anxiety” as keywords. In addition to conducting a systematic search in PubMed, we utilized the “related articles” feature to broaden our search scope and carefully evaluate the abstracts, studies, and citations. Moreover, our search in the databases was limited to records available in the English language.

### 2.2 Selection standards for included studies

The search and data collection for this meta-analysis were conducted in 2023. This comprehensive meta-analysis included case–control studies and cohort studies that determined the association of tinnitus with the progression and development of cognitive disorders. This study comprehensively includes published data from various periods, ensuring a comprehensive coverage of the available literature. This meta-analysis has included the research studies conducted in and after 2000, specifically chosen for their utilization of novel outcome variables and the delivery of authentic and reliable results.

To maintain the exclusion criteria, the following points were taken into consideration and applied during the study selection process: (1) exclude animal research, (2) restrict case reports, and studies with insufficient and unpublished data for meta-analysis, (3) exclude articles without proper methodology and outcomes, (4) exclude derivative data sources, such as review articles, (5) exclude studies having pediatric populations or individuals without a confirmed diagnosis of tinnitus, and (6) participants with tinnitus who also had concurrent life-threatening conditions such as cancer and cardiac disorders were excluded from the study to ensure a focused analysis on the specific impact of tinnitus on health outcomes. A broad summary of the characteristics of all the included studies is presented in Table 1.

**Table 1:** Summary of characteristics of all studies included in the present analysis.

**Sr no**.	**Author/ country**	**Year**	**Study type**	**Sample size (case/ control)**	**Gender**	**Age (years)**	**Follow-up period**	**Outcome measures**	**Evaluation**	**Reference**
1	Cheng et al./ Taiwan	2021	Case–control study	2,616 (1308/1308)	Women and men	30–64	10 years	Data obtained from Taiwan National Health Insurance Research Database	In patients with tinnitus, the risk of developing dementia increases up to 60–70%	Cheng et al., 2021
2	Hallam et al./ UK	2004	Case–control study	92 (43/49)	Women and men	20–60	–	STAI, CFQ	Tinnitus patients, as compared to healthy individuals, have cognitive difficulties, learning attention deficit issues, and anxiety attacks	Hallam et al., 2004
3	Langenbach et al./ Germany	2005	Cohort study	44 early diagnosed tinnitus patients	Women and men	More than 18	8 months	SCL 90-R	Tinnitus patients are highly prone to develop psychological problems and sleeping disorder	Langenbach et al., 2005
4	Te Chu et al./ Taiwan	2020	Cohort study	37,971 (12,657/25,314)	Women and men	41–64	10 years	Data were retracted from the Taiwan National Health Insurance Research Database	In tinnitus patients, a significant risk of developing Alzheimer's and Parkinson's disorders was found	Chu et al., 2020
5	Golub et al./ USA	2017	Cohort study	1,881 (204/ 1,677)	Women and men	>65	20 years	Data were retracted from an ongoing prospective cohort population-based study project	Hearing loss increases the risk of dementia	Golub et al., 2017
6	Deal et al./ USA	2017	Cohort study	3075	Women and men	70–79	7 years	Data were retracted from an ongoing cohort study	Results found that in older aged people, impaired hearing increased the risk of dementia profoundly	Deal et al., 2017
7	Zarenoe et al./ Sweden	2017	Case–control study	100 (50/50)	Women and men	60–80	-	PSQI	Patients suffering from tinnitus have experienced poor sleep quality and insomnia	Zarenoe et al., 2017
8	Pierce et al./ USA	2011	Clinical trial sub-group study (NCT00567892)	14	Women and men	18–60	-	COWAT, CVLT	Tinnitus patients showed significant learning problems and weak memory	Pierce et al., 2012
9	Loprinzi et al./ USA	2013	Cross-sectional study	696	Women and men	70–85	-	PHQ-9	Outcomes showed that in tinnitus patients, depression illness has a high significance	Loprinzi et al., 2013
10	Park et al./ South Korea	2022	Cross-sectional study	5,129	Women and men	>60	-	Data were retracted from an ongoing survey on a large scale by the Korean Centers for Disease Control and Prevention. Analysis	The older group of tinnitus patients found poor quality of life and high severity of depression	Park et al., 2022
11	Lasisi et al./ Nigeria	2010	Cohort study	1302	Women and men	>65	6 years	WHOQoL-Bref, PHQ-9	Chronic tinnitus in elderly patients is associated with impaired psychological and mental health, induced learning and auditory attention disorder, and depression	Lasisi et al., 2010
12	Gopinath et al./ USA	2010	Cohort study	1,214	Women and men	>60	5 years	SF-36	Tinnitus patients have a high risk of psychological distress, depression, and dizziness	Gopinath et al., 2010
13	Aazh et al./ UK	2017	Cross-sectional study	184	Women and men	>60	-	HADS	In the elderly tinnitus, patient's prevalence of insomnia and depression was found highly significant	Aazh et al., 2017
14	Fetoni et al./ Rome	2021	Cross-sectional study	102	Women	>60	-	HADS	In female sex, tinnitus has been associated with highly significant altered psychological distress and cognitive impairment	Fetoni et al., 2021
15	Park et al./ South Korea	2021	Cross-sectional study	10,979	Women and men	>40	-	Data were retracted from an ongoing survey on a large-scale by the Korean Centers for Disease Control and Prevention. Analysis performed by PHQ-9	Results revealed that in the elderly and middle-aged tinnitus patients' inducement of depression is more significant	Park et al., 2021
16	Davies et al./ UK	2017	Cohort study	7,685	Women and men	>60	11 years	IQCODE	Elderly patients with hearing impairment are more likely to suffer from dementia	Davies et al., 2017
17	Koning et al./ Netherlands	2019	Cohort study	165	Men	>60	2 years	Questionnaire	Tinnitus patients suffering from chronic tinnitus experienced sleep difficulty and insomnia	Koning, 2019

STAI, state trait anxiety inventory; CFQ, cognitive failures questionnaire; CVLT, California verbal learning test; COWAT, controlled oral word association test; SCL 90-R, The Symptom CheckList-90-Revised; PSQI, Pittsburgh Sleep Quality Index; PHQ-9, Patient Health Questionnaire-9; WHOQoL-Bref, World Health Organization Quality of Life Brief Version; SF-36, Short Form 36-item Health Survey; HADS, Hospital Anxiety and Depression Scale; IQCODE, Informant Questionnaire on Cognitive Decline in the Elderly.

For data retrieval and salvage, the following parameters were taken into consideration: (1) case–control and cohort studies reporting cognitive impairments (e.g., memory loss and attention deficits) and psychological impairments (e.g., anxiety and depression) associated with tinnitus were considered while collecting data, (2) studies with adequate population size, (3) studies with verified measuring methods and statistical analysis, (4) studies with a population including both men and women, (5) studies with an adult population (18 years and above), and (6) research studies with proper analyses and findings were included.

### 2.3 Description of outcome measures

The State-Trait Anxiety Inventory (STAI) is the outcome measure used to assess anxiety levels and their impact on cognitive functioning. It has been designed to measure both state and trait anxiety. It can be utilized in cognitive impairment detection to assess the impact of anxiety on cognitive functioning (Thomas and Cassady, 2021). The Cognitive Failures Questionnaire (CFQ) is a key tool for understanding the subjective cognitive difficulties of individuals with cognitive impairments. It is employed to measure memory and perception (Volosin et al., 2023). The Symptom Checklist-90-Revised (SCL 90-R) is used for the evaluation of a broad spectrum of psychological symptoms, including anxiety, depression, and attention deficit or obsessive compulsiveness (Shafique et al., 2017). The Pittsburgh Sleep Quality Index (PSQI) is valuable for assessing sleep quality and patterns. Sleep disturbances are often intertwined with cognitive issues, and PSQI provides a standardized approach to assess this critical aspect of wellbeing (Backhaus et al., 2002). Patient Health Questionnaire-9 (PHQ-9) is crucial for evaluating depressive symptoms in the context of cognitive impairments. Its inclusion aids in identifying and quantifying depressive features (Kroenke et al., 2001). The World Health Organization Quality of Life Brief Version (WHOQoL-Bref) serves as a vital measure in cognitive impairment research, offering a comprehensive assessment of the overall quality of life (Whoqol Group, 1998). The Short Form 36-item Health Survey (SF-36) is pivotal in cognitive impairment studies, providing a standardized approach to assess physical and mental health (Brazier et al., 1992). The California Verbal Learning Test (CVLT) is a comprehensive verbal assessment that evaluates auditory attention, learning, and memory functions through a multifactorial approach (Delis et al., 1988). The Hospital Anxiety and Depression Scale (HADS) is an invaluable tool for researchers to examine cognitive impairments and conduct concurrent assessments of anxiety and depression (Bocéréan and Dupret, 2014). The Informant Questionnaire on Cognitive Decline in the Elderly (IQCODE) is particularly relevant in studies involving older populations, providing an informant-based perspective on cognitive decline. Its use enhances the comprehensive assessment of cognitive impairments, especially in research focused on aging and dementia (Mathew et al., 2022). The Controlled Oral Word Association Test (COWAT) assesses verbal fluency and executive functions by measuring an individual's ability to generate words, providing valuable insights into cognitive outcomes (Ross et al., 2007).

These measures collectively contribute to a comprehensive assessment of cognitive functioning, emotional wellbeing, sleep quality, and overall quality of life, which are relevant aspects in the evaluation of neurodegenerative and cognitive disorders such as Parkinson's disease and dementia.

### 2.4 Extraction of data

**Figure 1:**
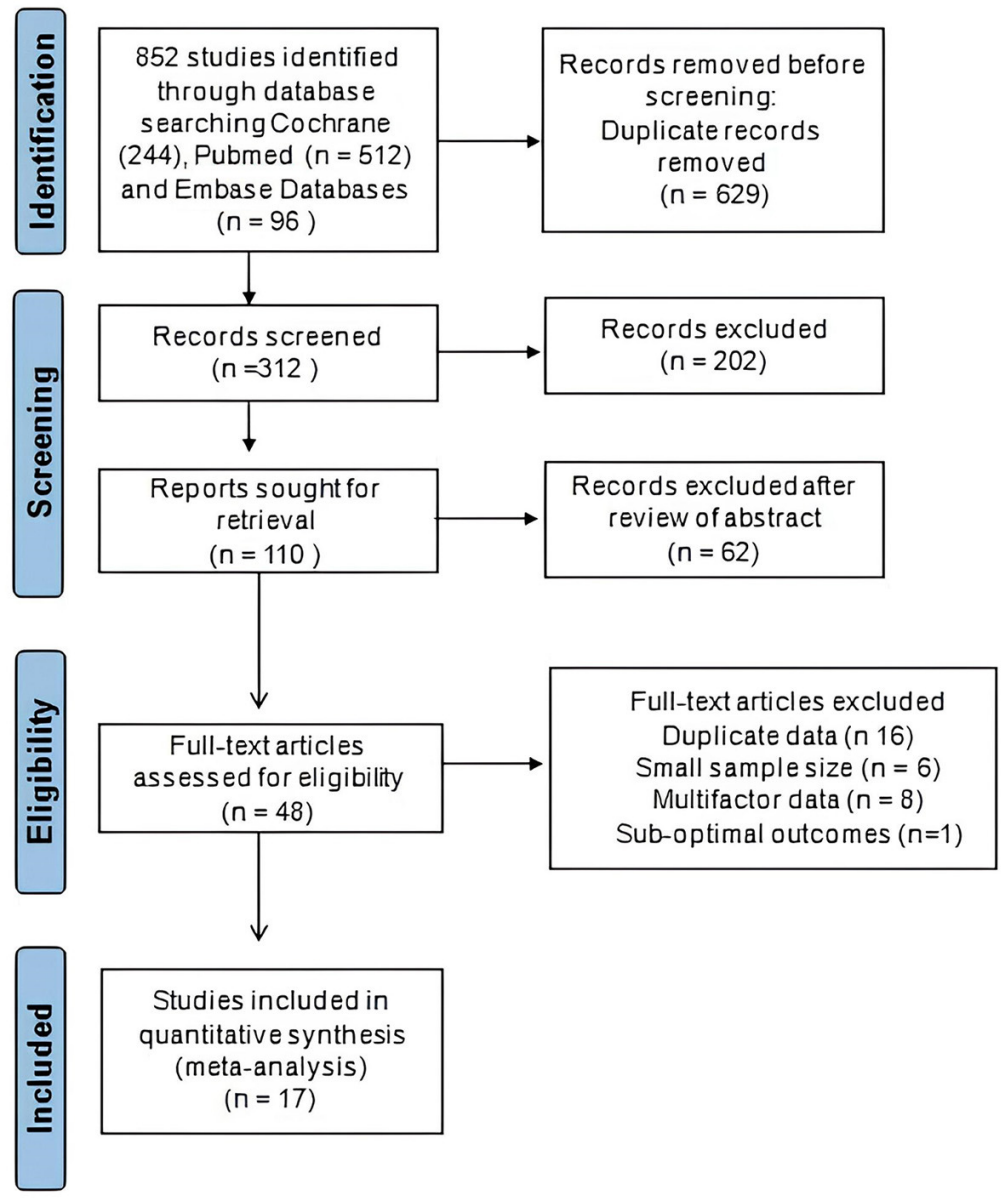
Systemic PRISMA flowchart for meta-analysis.

Two distinct investigators independently extracted the data. The data from the included studies were systematically gathered and organized in a standardized data collection spreadsheet on Excel. The included studies provided the following information, which was gathered and compiled for analysis: author name, location, publication year, sample size, gender, age, follow-up period, measuring outcomes, and association of tinnitus with the incidence and progression of cognitive disorders. The process of study selection is visually depicted in the PRISMA flowchart presented in Figure 1. We extracted the odd ratio (OR) and 95% confidence interval (95% CI) from the data presented in research studies to assess the potential risk of developing cognitive disorders associated with tinnitus. Moreover, we arranged the collected data and categorized it on the basis of age, which analyzes the potential variations or trends in the relationship between tinnitus and cognitive impairments across different demographic groups.

A Microsoft Excel spreadsheet was created to serve as a comprehensive database for our meta-analysis, comprising all relevant data for analysis. Decisions were compared, and disagreements were resolved by consulting another review author. Any kind of language restriction was not implemented.

### 2.5 Statistical analyses

For all kinds of statistical analyses in this meta-analysis study, we used Review Manager, Version 5.3 (Cochrane Collaboration, Oxford, England). This study was conducted following the guidelines set forth by the Preferred Reporting Items for Systematic Reviews and Meta-Analyses (PRISMA) protocol. ORs of included research studies were extracted to determine the association between tinnitus and the risk of cognitive disorders by a random-effects model. The pooled OR and corresponding 95% confidence interval (CI) were calculated to estimate the incidence of cognitive disorders in patients with tinnitus.

Heterogeneity among the studies was assessed using both the chi-square (χ^2^) test and the I^2^ statistic. If Cochran's Q-test yields a *P-*value below 0.10, it indicates statistically significant heterogeneity among the studies being analyzed.

## 3 Results

### 3.1 Description of included studies

We have included 17 studies in this meta-analysis, including both case–control trials and cohort studies. Six studies out of 17 have a population age of 18–60 years, and 11 include a patient population > 60 years old. Moreover, only two studies have a population size of < 100. In all included studies, the risk of cognitive impairments, including dementia, auditory attention and learning, poor sleep quality, anxiety, and depression, was determined in the patients who suffered from tinnitus. In Table 1, the characteristics and findings of the included studies are summarized.

In the case–control trials, outcome measures such as STAI, CFQ, COWAT, CVLT, PSQI, PHQ-9, SCL 90-R, WHOQoL-Bref, SF-36, HADS, and IQCODE, were used for analysis. The current comprehensive meta-analysis was performed, involving a meticulous literature search that identified and included 17 research studies. In the first step, a total of 852 studies were identified via database searching. After removing duplicates and conducting a preliminary screening of full-text articles, a total of 48 articles were assessed for eligibility. Out of these, 31 studies were excluded based on criteria such as duplicate data, limited sample size, unreliable and complex data, poor assessment methodology, and sub-optimal outcomes. The study selection is illustrated in a PRISMA flow diagram (Figure 1).

### 3.2 Bias risk

The Cochrane Collaboration independently evaluated the potential bias risk for each included study to assess methodological quality. Several research trials showed a high risk, with major sources of bias associated with the appropriateness of randomization, allocation concealment, random sequence generation, and protection from other potential biases. Overall, a moderate risk of bias was identified across the studies, indicating some variability in methodological quality. Supplementary Figure S1 reveals significant variability in study quality, with some studies demonstrating low risk in most domains. However, several studies exhibited limitations, particularly in allocation concealment and blinding.

### 3.3 Association of tinnitus with cognitive disorders

**Figure 2:**
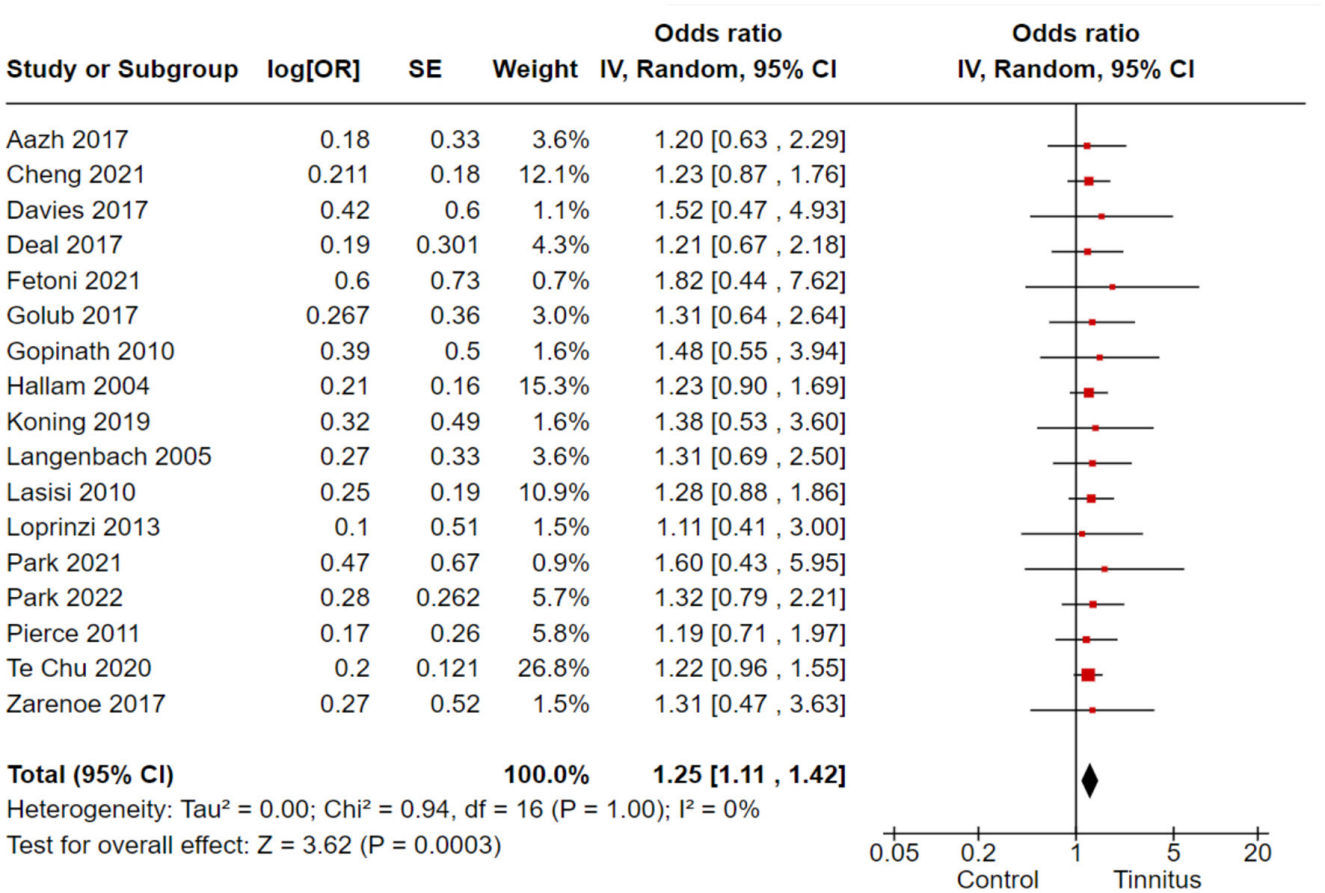
Correlation of tinnitus with the inducement of cognitive disabilities.

In the correlation analysis between tinnitus and cognitive disorders, we employed the COWAT, CFQ, WHOQoL-Bref, and SF-36 as the outcome variables to assess cognitive functions in tinnitus patients. A total of 17 cohort and randomized control studies with 62,270 subjects reported adequate data to produce a pooled estimation of the study effect size for tinnitus association with the inducement of cognitive disorders: OR 1.25, 95% CI: 1.11–1.42, *P* = 0.003; heterogeneity test; I^2^ = 0%, Chi^2^ = 0.94 (Figure 2).

### 3.4 Association of tinnitus with dementia

**Figure 3:**
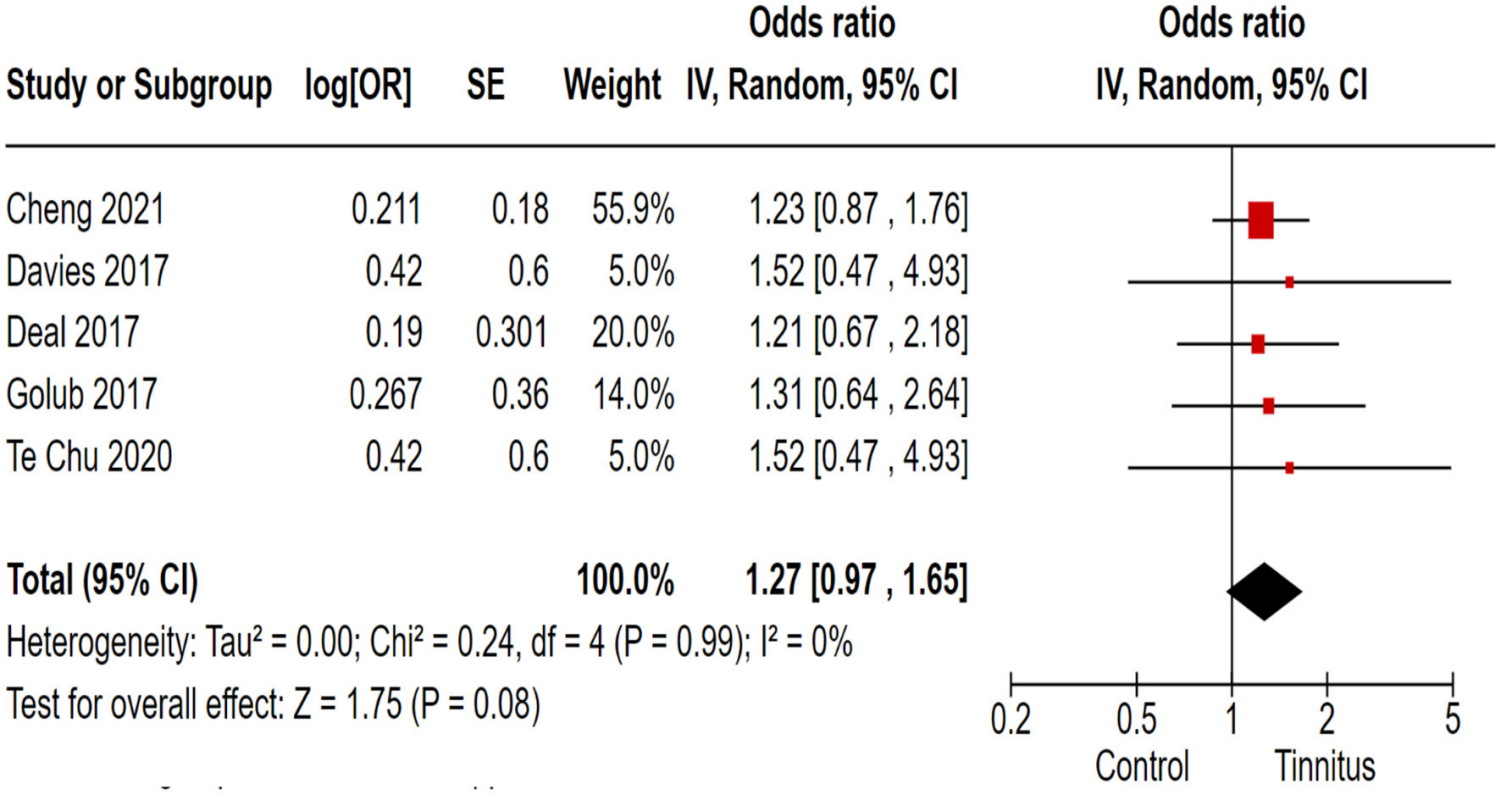
Correlation of tinnitus with the inducement of dementia.

In the correlation analysis between tinnitus and dementia, we employed the IQCODE as the outcome variable to assess dementia in tinnitus patients. Out of 17 trials, 5 cohort and randomized control studies reported adequate data to produce a pooled estimation of the study effect size for tinnitus association with the inducement of dementia: OR 1.27, 95% CI: 0.97–1.65, *P* = 0.08; heterogeneity test; I^2^ = 0%, Chi^2^ = 0.24 (Figure 3).

### 3.5 Association of tinnitus with learning and auditory attention

**Figure 4:**
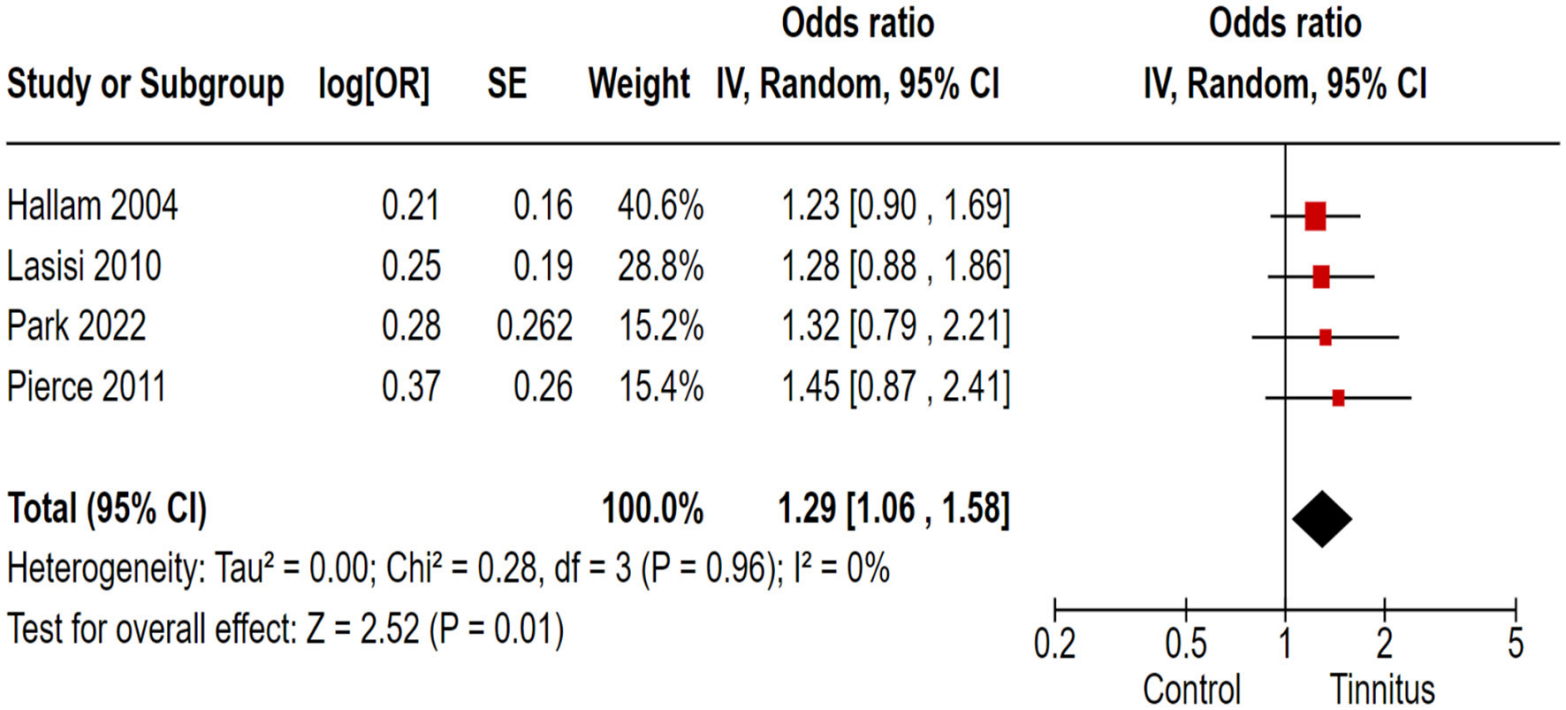
Correlation of tinnitus with the learning and auditory attention deficit.

In the correlation analysis between tinnitus and learning disorders, CVLT and COWAT were used as outcome variants, and researchers retracted the relevant data from ongoing large-scale population-based cross-sectional studies. Out of 17 trials, 4 cohort and randomized control studies reported adequate data to produce a pooled estimation of the study effect size for tinnitus association with the inducement of learning and auditory attention defects: OR 1.29, 95% CI: 1.06–1.58, *P* = 0.01; heterogeneity test; I^2^ = 0%, Chi^2^ = 0.28 (Figure 4).

### 3.6 Association of tinnitus with poor sleep quality

**Figure 5:**
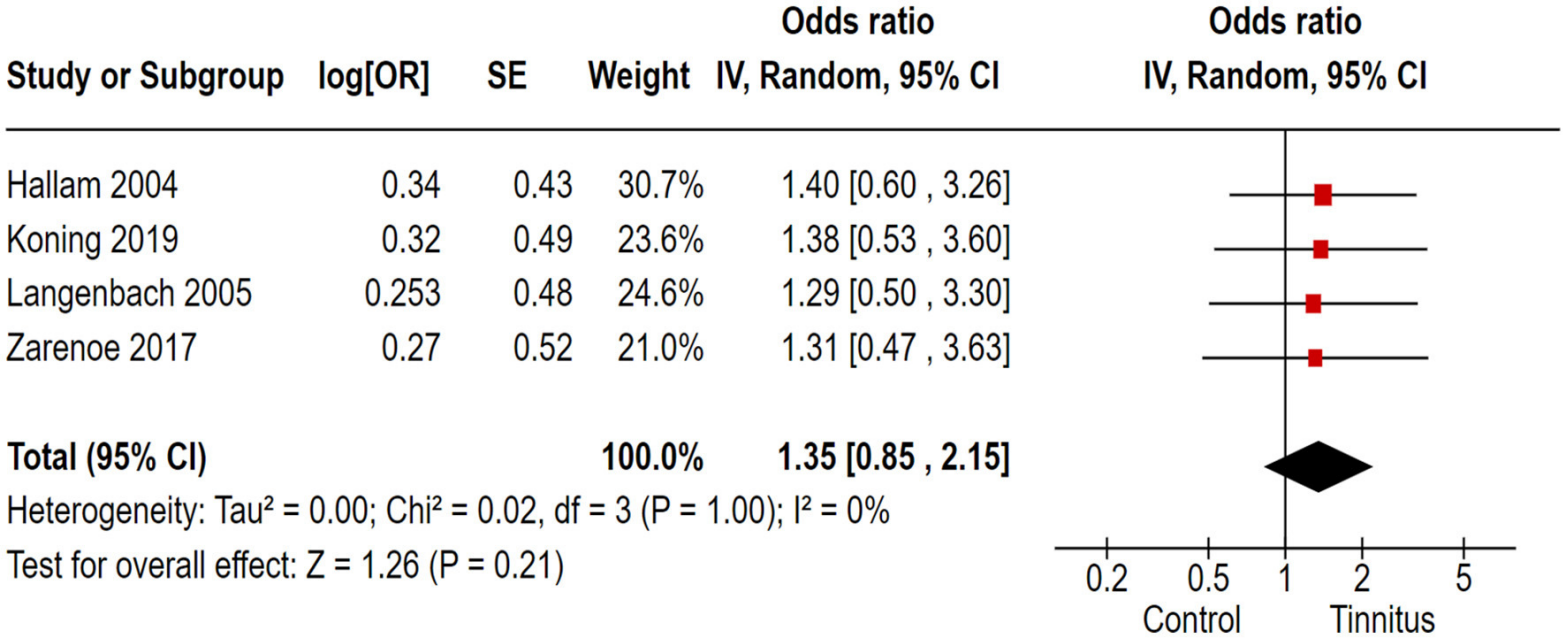
Correlation of tinnitus with the inducement of sleep disturbances.

In the correlation analysis between tinnitus and sleep quality, we employed the PSQI as the outcome variable to assess sleep quality in tinnitus patients. Out of 17 trials, 4 cohort and randomized control studies reported adequate data to produce a pooled estimation of the study effect size for tinnitus association with the inducement of poor sleep quality: OR 1.35, 95% CI: 0.85–2.15, *P* = 0.21; heterogeneity test; I^2^ = 0%, Chi^2^ = 0.02 (Figure 5).

### 3.7 Association of tinnitus with anxiety

**Figure 6:**
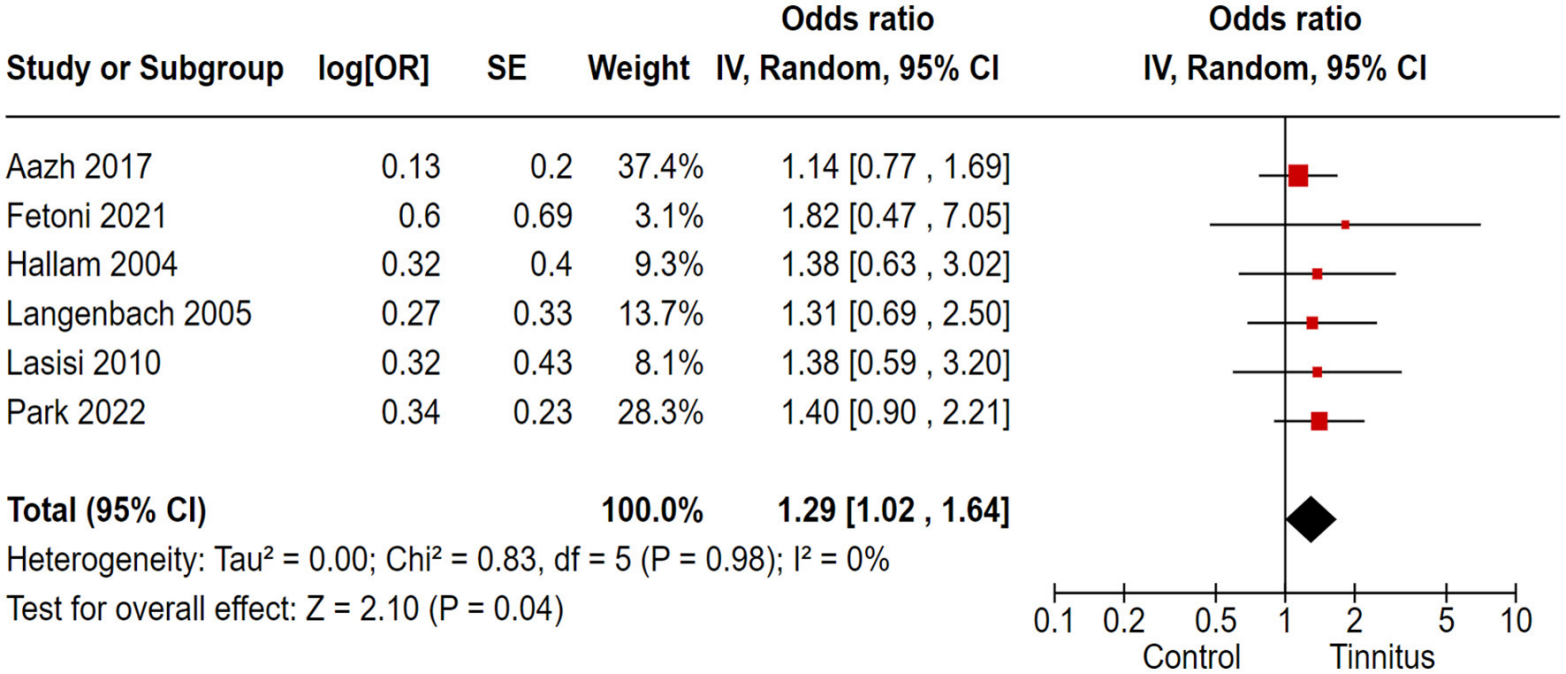
Correlation of tinnitus with the inducement of anxiety.

In the correlation analysis between tinnitus and anxiety, we employed the STAI, HADS, and SCL 90-R as the outcome variables to assess anxiety in tinnitus patients. Out of 17 trials, 6 cohort and randomized control studies reported adequate data to produce a pooled estimation of the study effect size for tinnitus association with the inducement of anxiety: OR 1.29, 95% CI: 1.02–1.64, *P* = 0.04; heterogeneity test; I^2^ = 0%, Chi^2^ = 0.83 (Figure 6).

### 3.8 Association of tinnitus with depression

**Figure 7:**
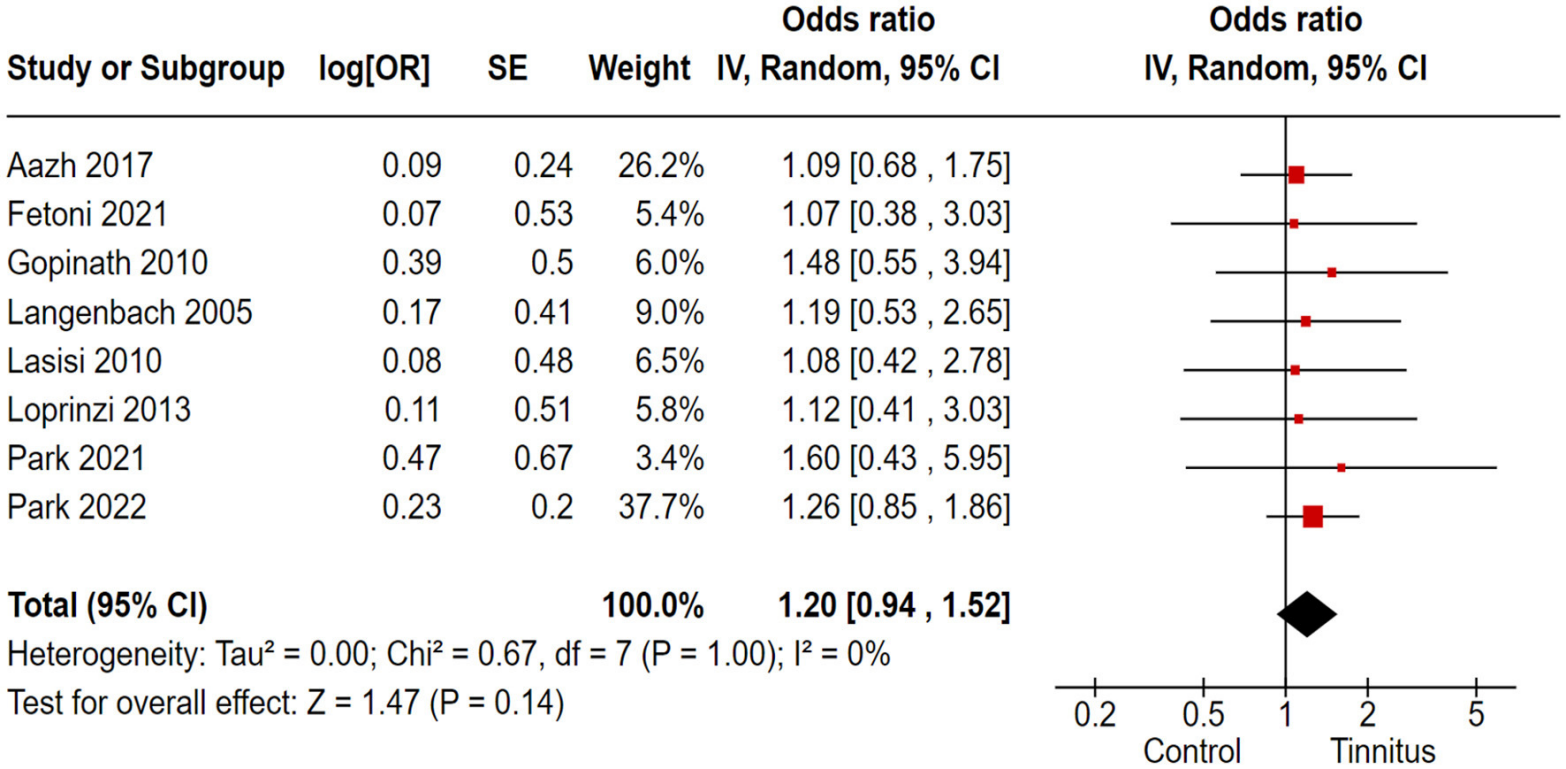
Correlation of tinnitus with the inducement of depression.

In the correlation analysis between tinnitus and depression, we employed the PHQ-9, HADS, and SCL 90-R as the outcome variables to assess depression in tinnitus patients. Out of 17 trials, 8 cohort and randomized control studies reported adequate data to produce a pooled estimation of the study effect size for tinnitus association with the inducement of depression: OR 1.20, 95% CI: 0.94–1.52, *P* = 0.14; heterogeneity test; I^2^ = 0%, Chi^2^ = 0.67 (Figure 7).

### 3.9 Association of tinnitus with cognitive disorders in patients 18–60 years old

**Figure 8:**
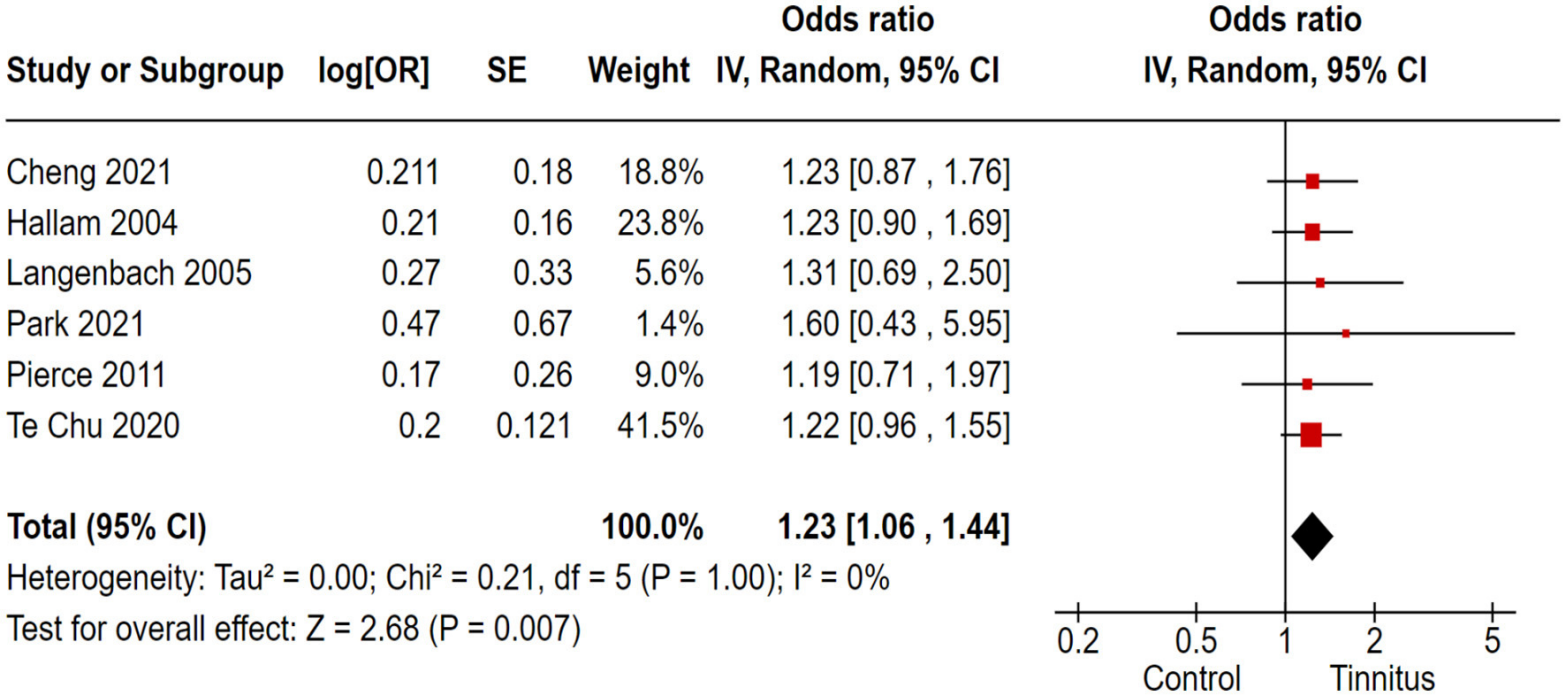
Correlation of tinnitus with the inducement of cognitive disorders in 18- to 60-year-old study population.

Out of 17 trials, 6 cohort and randomized control studies reported adequate data to produce a pooled estimation of the study effect size for tinnitus association with the inducement of cognitive impartments in the 18–60-year-old study population; OR 1.23, 95% CI: 1.06–1.44, *P* = 0.007; heterogeneity test; I^2^ = 0%, Chi^2^ = 0.21 (Figure 8).

### 3.10 Association of tinnitus with cognitive disorders in patients 60 years and older

**Figure 9:**
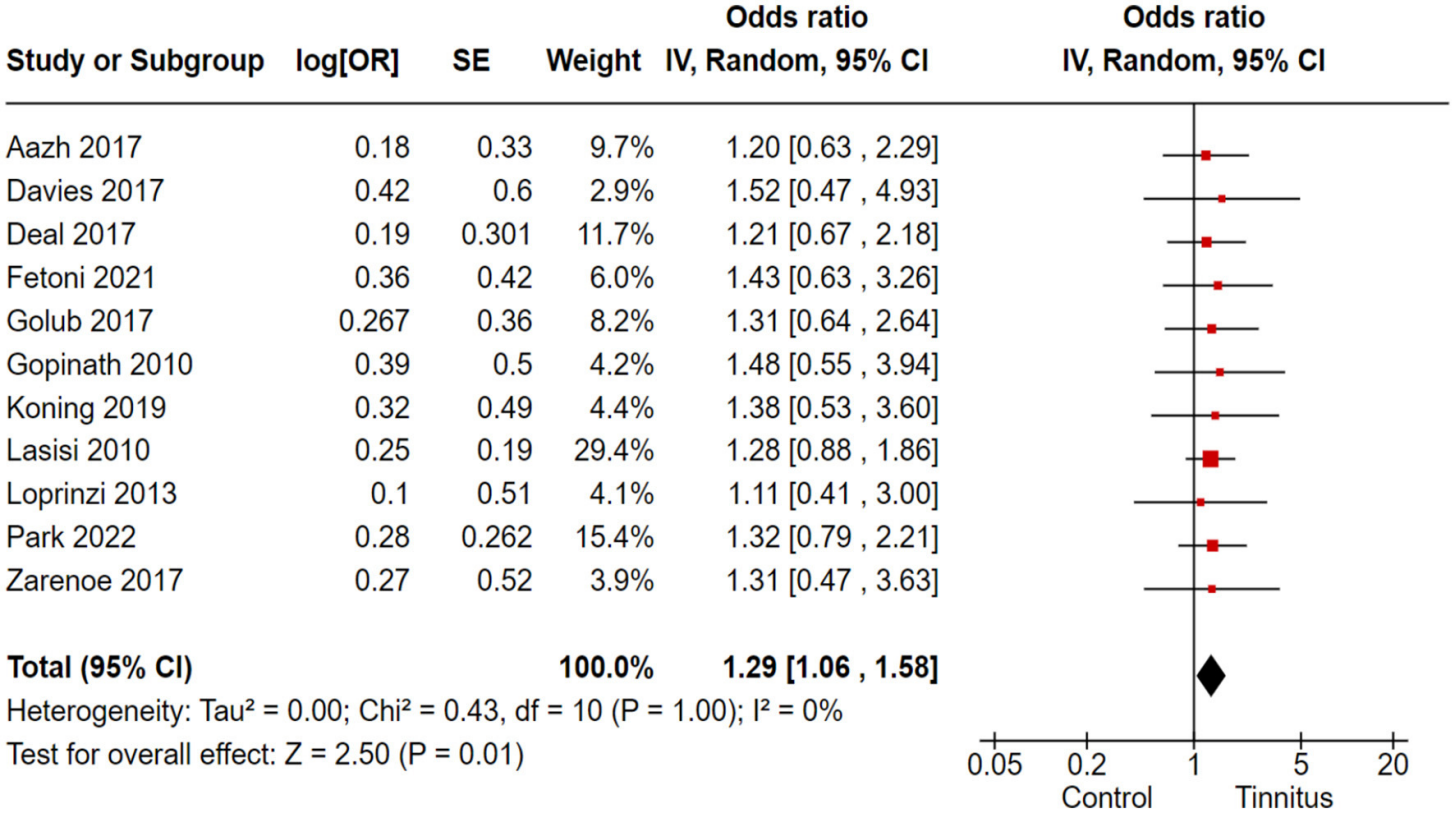
Correlation of tinnitus with the inducement of cognitive disorders in > 60-year-old study population.

Out of 17 trials, 11 cohort and randomized control studies reported adequate data to produce a pooled estimation of the study effect size for tinnitus association with the inducement of cognitive disorders in the > 60-year-old study population: OR 1.29, 95% CI: 1.06–1.58, *P* = 0.01; heterogeneity test; I^2^ = 0%, Chi^2^ = 0.43 (Figure 9).

## 4 Discussion

This comprehensive review meta-analysis study included 17 studies with 62,270 study participants, which provided data for analysis. The estimated pooled analysis outcomes of our study revealed that tinnitus has a strong association with the inducement of dementia, anxiety, altered learning and auditory attention, depression, and sleep disruption in tinnitus patients, especially elderly patients. Tinnitus has been identified as an independent risk factor for both dementia and Parkinson's disease (Chu et al., 2020). Moreover, multiple research studies have consistently concluded that there is a notable increase in the incidence and progression of dementia among individuals with tinnitus (Deal et al., 2017; Golub et al., 2017).

In this comprehensive meta-analysis, for the first time, we have established a correlation between tinnitus and cognitive impairments by analyzing population-based cohort studies and randomized case–controlled studies. Our investigation focused on the association of tinnitus with the risk of cognitive distress, including dementia, altered learning attention, poor sleep quality, anxiety, and depression. Furthermore, in this study, we stratified the subjects into subgroups based on age. The pooled analysis findings of the tinnitus population revealed a significant association with several cognitive impairments, including dementia, impaired learning and auditory attention, anxiety, depression, and severe insomnia. Remarkably, we found an elevated risk of cognitive impairments in both the age groups of 18 and 60. However, when comparing the population aged > 60 (older patients) with those aged 18–60 years, it was found that the group of older patients suffering from tinnitus exhibited a notably stronger association with the development of cognitive disorders. These findings suggest that elderly patients are more susceptible to the onset of cognitive impairments.

In a previous meta-analysis review study, it was found that tinnitus disturbs cognitive functionality and significantly increases the risk of short-term memory loss or dementia (Clarke et al., 2020). Another meta-analysis study highlighted a strong association between tinnitus and cognitive disability. The findings indicated that individuals with tinnitus often faced concurrent conditions such as depression, poor sleep, and anxiety (Clarke et al., 2018). A recently published study has unveiled that elderly tinnitus patients face a considerably higher risk of dementia, and they also exhibit associations with other cognitive impairments, including anxiety, depression, and sleep difficulties (Malesci et al., 2021). However, contrary to expectations, an investigational study conducted in 2021 revealed a positive association between tinnitus and enhanced cognitive performance in non-Hispanic elderly individuals with hearing loss. These unexpected findings suggest that ethnicity plays a significant role in disease expression. Therefore, to further consolidate and comprehend such results, future investigations should incorporate longitudinal research studies for comprehensive validation and mechanistic insights (Hamza and Zeng, 2021).

To protect tinnitus patients from developing cognitive impairments, a comprehensive approach is crucial. The approach should include implementing effective tinnitus management with sound therapy and counseling; promoting a healthy lifestyle with regular exercise and a balanced diet; scheduling regular medical check-ups to monitor cognitive health; identifying potential issues early on; reviewing medications with healthcare providers for potential cognitive side effects (Han et al., 2009), and empowering patients with educational support and resources to manage both tinnitus and cognitive health effectively (Kompis et al., 2004). Further advanced research studies are required in this field to provide a more comprehensive understanding of the association between tinnitus and cognitive impairments.

Our findings indicated a strong association between tinnitus and the risk of cognitive disabilities. However, there are some study limitations in this meta-analysis, including variations in follow-up periods, diverse analytical measuring methods, questionnaires across studies, the absence of methodological quality assessments for the included studies, and different population sizes.

## 5 Conclusion

In our meta-analysis study, we found a strong association between tinnitus and the risk of cognitive impairments in patients suffering from tinnitus. Moreover, it was evident that elderly tinnitus patients (>60 years old) exhibited a significantly higher susceptibility to developing cognitive impairments, particularly dementia, compared to younger individuals.

## Data availability statement

The original contributions presented in the study are included in the article/Supplementary material, further inquiries can be directed to the corresponding author.

## Author contributions

DY: Conceptualization, Formal analysis, Methodology, Writing—original draft. DZ: Conceptualization, Formal analysis, Methodology, Writing—original draft. XZ: Software, Validation, Writing—original draft. XL: Supervision, Writing—review & editing.

## Supplementary material

Supplementary Figure S1 Risk of bias of the included studies in the meta-analysis study.

Supplementary Table S1 Details of various parameters and criteria used in this study.

## References

[B1] Aazh H. Lammaing K. Moore B. C. J. (2017). Factors related to tinnitus and hyperacusis handicap in older people. Int. J. Audiol. 56, 677–684. 10.1080/14992027.2017.1335887 28625091

[B2] Alster J. Shemesh Z. Ornan M. Attias J. (1993). Sleep disturbance associated with chronic tinnitus. Biol. Psychiatr. 34, 84–90. 10.1016/0006-3223(93)90260-K 8373941

[B3] Backhaus J. Junghanns K. Broocks A. Riemann D. Hohagen F. (2002). Test-retest reliability and validity of the pittsburgh sleep quality index in primary insomnia. J. Psychosom. Res. 53, 737–740. 10.1016/S0022-3999(02)00330-6 12217446

[B4] Baguley D. McFerran D. Hall D. (2013). Tinnitus. Lancet 382, 1600–1607. 10.1016/S0140-6736(13)60142-7 23827090

[B5] Bocéréan C. Dupret E. (2014). A validation study of the Hospital Anxiety and depression scale (HADS) in a large sample of French employees. BMC Psychiatr. 14, 354. 10.1186/s12888-014-0354-0 25511175 PMC4476305

[B6] Brazier J. E. Harper R. Jones N. M. O'Cathain A. Thomas K. J. Usherwood T. . (1992). Validating the SF-36 health survey questionnaire: new outcome measure for primary care. BMJ 305, 160–164. 10.1136/bmj.305.6846.160 1285753 PMC1883187

[B7] Cheng Y. F. Xirasagar S. Yang T. H. Wu C. S. Kao Y. W. Lin H. C. . (2021). Risk of early-onset dementia among persons with tinnitus: a retrospective case-control study. Sci. Rep. 11, 13399. 10.1038/s41598-021-92802-y 34183724 PMC8238939

[B8] Chu H. T. Liang C. S. Yeh T. C. Hu L. Y. Yang A. C. Tsai S. J. . (2020). Tinnitus and risk of Alzheimer's and Parkinson's disease: a retrospective nationwide population-based cohort study. Sci. Rep. 10, 12134. 10.1038/s41598-020-69243-0 32699252 PMC7376045

[B9] Clarke N. A. Akeroyd M. A. Henshaw H. Hoare D. J. (2018). Association between subjective tinnitus and cognitive performance: protocol for systematic review and meta-analysis. BMJ Open 8, e023700. 10.1136/bmjopen-2018-023700 30104320 PMC6091911

[B10] Clarke N. A. Henshaw H. Akeroyd M. A. Adams B. Hoare D. J. (2020). Associations between subjective tinnitus and cognitive performance: systematic review and meta-analyses. Trends Hear. 24, 2331216520918416. 10.1177/2331216520918416 32436477 PMC7243410

[B11] Davies H. R. Cadar D. Herbert A. Orrell M. Steptoe A. (2017). Hearing impairment and incident dementia: findings from the english longitudinal study of ageing. J. Am. Geriatr. Soc. 65, 2074–2081. 10.1111/jgs.14986 28734053 PMC5637915

[B12] Deal J. A. Betz J. Yaffe K. Harris T. Purchase-Helzner E. Satterfield S. . (2017). Hearing impairment and incident dementia and cognitive decline in older adults: the health ABC study. The J. Gerontol. Biol. Sci. Med. Sci. 72, 703–709. 10.1093/gerona/glw069 27071780 PMC5964742

[B13] Delis D. C. Freeland J. Kramer J. H. Kaplan E. (1988). Integrating clinical assessment with cognitive neuroscience: construct validation of the California verbal learning test. J. Consult. Clin. Psychol. 56, 123–130. 10.1037/0022-006X.56.1.123 3346437

[B14] Fetoni A. R. Cesare T. D. Settimi S. Sergi B. Rossi G. Malesci R. . (2021). The evaluation of global cognitive and emotional status of older patients with chronic tinnitus. Brain Behav. 11, e02074. 10.1002/brb3.2074 34288570 PMC8413806

[B15] Gallo K. E. B. Corrêa C. C. Gonçalves C. G. O. Correia Baran J. B. Marques J. M. Zeigelboim B. S. . (2023). Effect of tinnitus on sleep quality and insomnia. Int. Arch. Otorhinolaryngol. 27, e197–e202. 10.1055/s-0041-1735455 37125358 PMC10147471

[B16] Golub J. S. Luchsinger J. A. Manly J. J. Stern Y. Mayeux R. Schupf N. . (2017). Observed hearing loss and incident dementia in a multiethnic cohort. J. Am. Geriatr. Soc. 65, 1691–1697. 10.1111/jgs.14848 28323321 PMC5555781

[B17] Gopinath B. McMahon C. M. Rochtchina E. Karpa M. J. Mitchell P. (2010). Risk factors and impacts of incident tinnitus in older adults. Annal. Epidemiol. 20, 129–135. 10.1016/j.annepidem.2009.09.002 20123163

[B18] Hallam R. S. McKenna L. Shurlock L. (2004). Tinnitus impairs cognitive efficiency. Int. J. Audiol. 43, 218–226. 10.1080/14992020400050030 15250126

[B19] Hamza Y. Zeng F. G. (2021). Tinnitus is associated with improved cognitive performance in non-hispanic elderly with hearing loss. Front. Neurosci. 15, 735950. 10.3389/fnins.2021.735950 34776845 PMC8581543

[B20] Han B. I. Lee H. W. Kim T. Y. Lim J. S. Shin K. S. (2009). Tinnitus: characteristics, causes, mechanisms, and treatments. J. Clin. Neurol. 5, 11–19. 10.3988/jcn.2009.5.1.11 19513328 PMC2686891

[B21] Jafari Z. Kolb B. E. Mohajerani M. H. (2019). Age-related hearing loss and tinnitus, dementia risk, and auditory amplification outcomes. Ageing Res. Rev. 56, 100963. 10.1016/j.arr.2019.100963 31557539

[B22] Kompis M. Neuner N. T. Hemmeler W. Häusler R. (2004). Therapeutische Umschau. Revue Ther. 61, 15–20. 10.1024/0040-5930.61.1.15 14997995

[B23] Koning H. M. (2019). Sleep disturbances associated with tinnitus: reduce the maximal intensity of tinnitus. The Int. Tinnitus J. 23, 64–68. 10.5935/0946-5448.20190018 31469531

[B24] Kroenke K. Spitzer R. L. Williams J. B. (2001). The PHQ-9: validity of a brief depression severity measure. J. Gen. Int. Med. 16, 606–613. 10.1046/j.1525-1497.2001.016009606.x 11556941 PMC1495268

[B25] Lan T. Cao Z. Zhao F. Perham N. (2020). The association between effectiveness of tinnitus intervention and cognitive function-a systematic review. Front. Psychol. 11, 553449. 10.3389/fpsyg.2020.553449 33488438 PMC7815700

[B26] Langenbach M. Olderog M. Michel O. Albus C. Köhle K. (2005). Psychosocial and personality predictors of tinnitus-related distress. Gen. Hosp. Psychiatr. 27, 73–77. 10.1016/j.genhosppsych.2004.08.008 15694221

[B27] Lasisi A. O. Abiona T. Gureje O. (2010). Tinnitus in the elderly: Profile, correlates, and impact in the Nigerian study of ageing. Otolaryngol. Head Neck Surg. Off. J. Am. Acad. Otolaryngol. Head Neck Surg. 143, 510–515. 10.1016/j.otohns.2010.06.817 20869560

[B28] Loprinzi P. D. Maskalick S. Brown K. Gilham B. (2013). Association between depression and tinnitus in a nationally representative sample of US older adults. Aging Mental Health 17, 714–717. 10.1080/13607863.2013.775640 23461284

[B29] Malesci R. Brigato F. Di Cesare T. Del Vecchio V. Laria C. De Corso E. Fetoni A. R. (2021). Tinnitus and neuropsychological dysfunction in the elderly: a systematic review on possible links. J. Clin. Med. 10, 1881. 10.3390/jcm10091881 33925344 PMC8123622

[B30] Mathew R. Sheetal S. Sauda P. Lekha C. Byju P. (2022). Utility of IQCODE (informant questionnaire on cognitive decline in the elderly) in diagnosing dementia in Malayalam speaking population. Neurol. India 70, 1947–1952. 10.4103/0028-3886.359172 36352592

[B31] Ost L. G. (2014). The efficacy of acceptance and commitment therapy: an updated systematic review and meta-analysis. Behav. Res. Ther. 61, 105–121. 10.1016/j.brat.2014.07.018 25193001

[B32] Paciello F. Ripoli C. Fetoni A. R. Grassi C. (2023). Redox imbalance as a common pathogenic factor linking hearing loss and cognitive decline. Antioxidants 12, 332. 10.3390/antiox12020332 36829891 PMC9952092

[B33] Park H. M. Jung J. Kim J. K. Lee Y. J. (2022). Tinnitus and Its association with mental health and health-related quality of life in an older population: a nationwide cross-sectional study. J. Appl. Gerontol. Off. J. Southern Gerontol. Soc. 41, 181–186. 10.1177/0733464820966512 33090056

[B34] Park M. Kang S. H. Nari F. Park E. C. Jang S. I. (2021). Association between tinnitus and depressive symptoms in the South Korean population. PloS ONE 16, e0261257. 10.1371/journal.pone.0261257 34928968 PMC8687527

[B35] Pattyn T. Van Den Eede F. Vanneste S. Cassiers L. Veltman D. J. Van De Heyning P. . (2016). Tinnitus and anxiety disorders: a review. Hearing Res. 333, 255–265. 10.1016/j.heares.2015.08.014 26342399

[B36] Pierce K. J. Kallogjeri D. Piccirillo J. F. Garcia K. S. Nicklaus J. E. Burton H. . (2012). Effects of severe bothersome tinnitus on cognitive function measured with standardized tests. J. Clin. Exp. Neuropsychol. 34, 126–134. 10.1080/13803395.2011.623120 22168528 PMC3313073

[B37] Ross T. P. Calhoun E. Cox T. Wenner C. Kono W. Pleasant M. . (2007). The reliability and validity of qualitative scores for the controlled oral word association test. Arch. Clin. Neuropsychol. Off. J. Nat. Acad. Neuropsychol. 22, 475–488. 10.1016/j.acn.2007.01.026 17317094

[B38] Savage J. Waddell A. (2014). Tinnitus. BMJ Clin. Evid. 2014:506. PMC4202663 25328113

[B39] Shafique N. Khalily M. T. Mchugh L. (2017). Translation and validation of symptom checklist-90. Pakistan J. Psychol. Res. 32, 545–561.

[B40] Thomas C. L. Cassady J. C. (2021). Validation of the state version of the state-trait anxiety inventory in a university sample. SAGE Open 11, 21582440211031900. 10.1177/21582440211031900

[B41] Volosin M. Hallgató E. Csábi E. (2023). Validation of the Hungarian version of the cognitive failures questionnaire (CFQ). Heliyon 9, e12910. 10.1016/j.heliyon.2023.e12910 36685410 PMC9853372

[B42] Whoqol Group (1998). Development of the world health organization WHOQOL-BREF quality of life assessment. The WHOQOL group. Psychol. Med. 28, 551–558. 10.1017/S0033291798006667 9626712

[B43] Zarenoe R. Hällgren M. Andersson G. Ledin T. (2017). Working memory, sleep, and hearing problems in patients with tinnitus and hearing loss fitted with hearing aids. J. Am. Acad. Audiol. 28, 141–151. 10.3766/jaaa.16023 28240981

