# Supplementary figures and images for: Tinnitus-associated cognitive and psychological impairments: a comprehensive review meta-analysis
Source: Front Neurosci. 2024 Feb 8;18:1275560. doi: 10.3389/fnins.2024.1275560 (PMC10881733; doi:10.3389/fnins.2024.1275560)

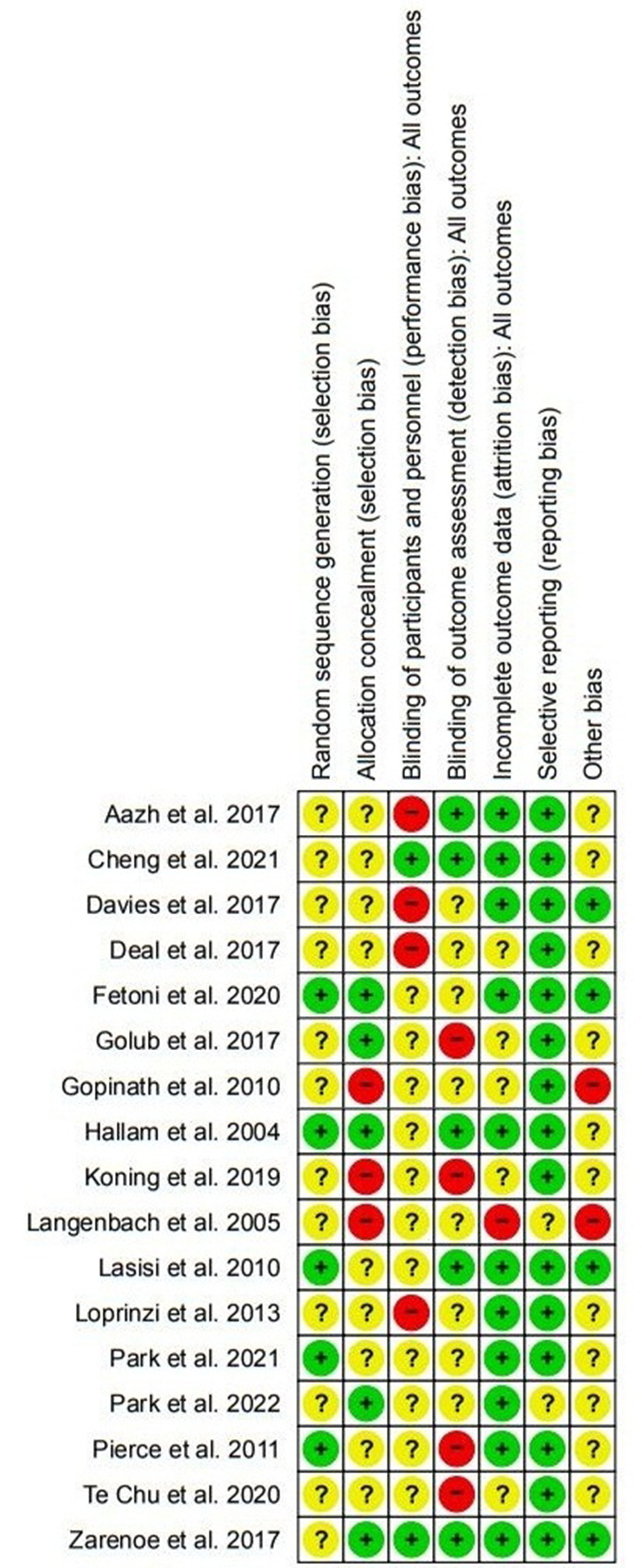

Supplement: Supplementary Figure S1 — Risk of bias of the included studies in the meta-analysis study. [file Image_1.JPEG]
